# Facile fabrication of microfluidic surface-enhanced Raman scattering devices via lift-up lithography

**DOI:** 10.1098/rsos.172034

**Published:** 2018-04-04

**Authors:** Yuanzi Wu, Ye Jiang, Xiaoshan Zheng, Shasha Jia, Zhi Zhu, Bin Ren, Hongwei Ma

**Affiliations:** 1 College of Biological Science and Engineering, Fuzhou University, Fuzhou 350002, People's Republic of China; 2 State Key Laboratory of Physical Chemistry of Solid Surfaces, the MOE Key Laboratory of Spectrochemical Analysis and Instrumentation, College of Chemistry and Chemical Engineering, and Collaborative Innovation Center of Chemistry of Energy Materials, Xiamen University, Xiamen 361005, People's Republic of China; 3 Suzhou Institute of Nano-Tech and Nano-Bionics, Chinese Academy of Sciences, Suzhou 215125, People's Republic of China

**Keywords:** lift-up lithography, surface-enhanced Raman scattering substrate, microfluidics

## Abstract

We describe a facile and low-cost approach for a flexibly integrated surface-enhanced Raman scattering (SERS) substrate in microfluidic chips. Briefly, a SERS substrate was fabricated by the electrostatic assembling of gold nanoparticles, and shaped into designed patterns by subsequent lift-up soft lithography. The SERS micro-pattern could be further integrated within microfluidic channels conveniently. The resulting microfluidic SERS chip allowed ultrasensitive *in situ* SERS monitoring from the transparent glass window. With its advantages in simplicity, functionality and cost-effectiveness, this method could be readily expanded into optical microfluidic fabrication for biochemical applications.

## 1. Introduction

Microfluidics has become increasingly attractive in chemical and biomolecular detection and analysis recently. It provides a precise manipulation of small amounts of biological samples with unique advantages such as low sample consumption, high throughput and high reaction efficiency [1,2]. Nowadays, microfluidics has been extensively applied in optical or spectroscopic analysis, such as UV-visible absorption, fluorescence, chemiluminescence and surface-enhanced Raman scattering (SERS). Among these techniques, SERS has become an emerging method for the detection of biomolecules with its high sensitivity and specificity [3]. Raman spectra can provide molecular fingerprints via the vibration spectra, which makes it possible to identify specific molecules from complex samples. However, Raman scattering suffers from low signal intensity due to the small scattering cross section. This problem can be overcome by using the SERS technique. When a molecule is attached or in proximity to the surface of the metallic nanostructures of Au, Ag or Cu, Raman signals of the molecules will be amplified by several orders of magnitude, and single-molecule sensitivity has been achieved for some molecules [4]. Therefore, SERS has emerged as a powerful tool for multiplex label-free detection with high sensitivity so far.

Great efforts have been made in constructing the SERS substrate inside the microfluidic channels, which may allow simultaneous monitoring of the reaction processes in a sensitive manner [5–11]. By using advanced top-down nanofabrication, such as electron beam lithography, the SERS substrate could be prefabricated and further embedded into a microfluidic channel [12,13]. Alternatively, SERS substrates could also be fabricated inside microfluidic channels directly by laser-induced nanoparticle growth [14,15] or thermal deposition [16,17]. However, most of these methods suffered from low enhancement, requiring special instruments, or poor surface uniformity. One of the common routes for fabricating SERS substrates is self-assembly of gold nanoparticles on an amino-modified surface. This method has been proved to be a robust, cost-effective strategy to produce SERS substrates with high enhancement and good uniformity over a large area. Such properties are very important for practical applications to achieve good reliability and reproducibility for the detection of small amounts of analytes [18,19].

The soft lithography method has been widely used in producing patterns of nanoparticles over large areas at the micrometre scale, such as pattern transfer with a polydimethylsiloxane (PDMS) stamp via the micro-contact printing method [20–26]. However, there are still several disadvantages to date for this method to produce a micro-patterned SERS substrate. For example, any impurities inherited from the transfer process or unnecessary contact may produce an interfering signal to the signal of the target species. Moreover, the stress experienced during the transfer procedure may destroy the evenly distributed and closely packed nanostructure of nanoparticles, which may lead to the decrease of the local enhancement of the ‘hot spot' by a subtle change of gap distance inside nano-assemblies.

To overcome the limitations of stamp transferring in soft lithography, in this work we transformed the conventional contact printing process to the lift-up lithography strategy [27]. The PDMS stamp removes the nanoparticles by contact and the untouched regions remain intact for further SERS detection. This method facilitates the fabrication process to obtain the layer of gold nanoparticles by self-assembly with a high-resolution two-dimensional structure without a complicated treatment. With this approach, we successfully fabricated two-dimensional structures of gold nanoparticles with a line width of several micrometres. We further bonded a PDMS cover with designed concave channels to the glass slide to make a microfluidic chip with an integrated SERS substrate. On-site SERS detection can be performed by directly focusing the incident laser onto the inner surface of the glass slide directly through the rear of the slide. In this way, we can avoid focusing through the PDMS and liquid layer, which may produce an interfering signal and reduce the collection efficiency. This facile approach exhibits the capability for disposable microfluidic SERS detection of analytes for a wide range of reactions.

## 2. Material and methods

### 2.1. Chemicals and materials

Chloroauric acid, trisodium citrate dihydrate, hydroxylamine hydrochloride, H_2_SO_4_, H_2_O_2_, NaOH, NaCl and other frequently used reagents were obtained from Sinopharm Chemical Reagent Co., Ltd. (3-Aminopropyl)-trimethoxysilane (APTMS), mercaptoethanol, Rhodamine 6G and Nile blue were purchased from Aldrich. The Sylgard 184 silicone elastomer kit (Dow Corning) was used to form PDMS. The DNA sequences of the oligonucleotides used in the study were DNA1: 5′ -TAMRA-ACACCGATC-(CH2)3-SH-3′ (DNA 1) and DNA2: 5′ -GATCGGGTGTGGGTGGCGTAAAGGGAGCATCGGACA-3′ (DNA 2) synthesized by Synbio Tech. (China). All the chemicals were used as received without further purification. Milli-Q deionized water was used throughout the studies.

### 2.2. Preparation of surface-enhanced Raman scattering-active surface

As a first step to fabricate gold nanoparticle-covered glass slides, uniform 60 nm gold nanoparticles were synthesized by the hydroxylamine seed-mediated method [28]. The cover glass was cleaned in piranha solution (35% H_2_O_2_ aqueous solution : concentrated H_2_SO_4_, 3 : 7/v : v) for 30 min, further rinsed in water and dried in a stream of nitrogen gas. The glass slide was placed in 0.2% APTMS aqueous solution for 24 h, followed by rinsing with water and drying to give an amino-modified surface. Then the glass slide was immersed in the gold colloidal suspension overnight to obtain the SERS substrate with self-assembled gold nanoparticles. The as-prepared surface was kept at 4°C before further treatment. In addition, the gold nanoparticles could also self-assemble on the different substrates, such as ITO-coated glass, silicon wafer and piranha solution-treated PDMS sheet.

### 2.3. Procedure of lift-up lithography of gold nanoparticles and fabrication of microfluidic chips

Master moulds for lift-up lithography and microfluidic chips were fabricated by conventional photolithography and the PDMS casting method. The micro-pattern of the photomask was transferred to a 50 µm thick SU8 photoresist cast on a silicon wafer via UV exposure. Then a degassed 10 : 1 mixture of a PDMS precursor and the curing agent was cast on the patterned silicon wafer. After the PDMS stamp was cured for 30 min at 70°C, it was lifted off and then aligned and sealed compactly on the glass slide coated with gold nanoparticles. Adhesion between the two surfaces was accomplished by further curing them for another 15 min at 70°C. Then, the PDMS stamp was peeled from the substrate, resulting in a surface with a gold nanoparticle micro-pattern complementary to that of the PDMS stamp. An as-prepared PDMS fluid layer was treated with oxygen plasma for 1 min, and then bound to the above-patterned surface to form the SERS substrate-embedded microchannels. The inlets and outlets were punched before the plasma process.

### 2.4. Surface-enhanced Raman scattering measurement

Normal Raman and SERS spectra were collected on the XploRA (Jobin-Yvon) system with a wavelength of 638 nm unless otherwise stated. A laser spot approximately 2 µm in diameter was obtained by using a 50× objective (NA 0.55, BD). The typical acquisition time was 10 s unless otherwise stated. The step length for line scanning is 1 µm.

For detection with Nile blue, 10^−4^ M Nile blue solution was injected into the microfluidic SERS chips and incubated for 1 h, followed by rinsing with water to remove the non-specifically bound molecules. SERS spectra were acquired from the glass slide by focusing the laser on the gold nanoparticle layer. The acquisition time was 10 s and the laser power was approximately 3 mW.

For detection of ochratoxin A (OTA), DNA was first dissolved in 10 mM PBS buffer containing 1 mM MgCl_2_ and stored at 4°C before use. Stoichiometric amounts of DNA 1 and 2 were mixed, heated to 94°C for 5 min and then slowly lowered to room temperature to form double-strain oligonucleotides. The oligonucleotides were incubated with the SERS substrate for 5 h. Then, 1 mM mercaptoethanol was injected and incubated for 10 min to occupy the remaining adsorption sites and keep the strands upright onto the gold surface. The unbound DNA strands were removed by rinsing with a PBS solution. The substrate was further incubated with OTA solutions followed by rinsing with pure water to remove non-specifically bound species. The SERS spectra were acquired with a 638 nm laser and a 50× objective. The acquisition time was 60 s and the laser power was approximately 120 µW. All the incubating and measuring processes were performed at a temperature of 20°C.

### 2.5. Characterization

The morphology of the gold nanoparticle-patterned surface was characterized by SEM (Nova, NanoSEM 230), and dark-field microscopy (XploRa, Horiba Jobin Yvon) using a 50× dark-field objective (NA = 0.5).

## 3. Results

**Scheme 1.**
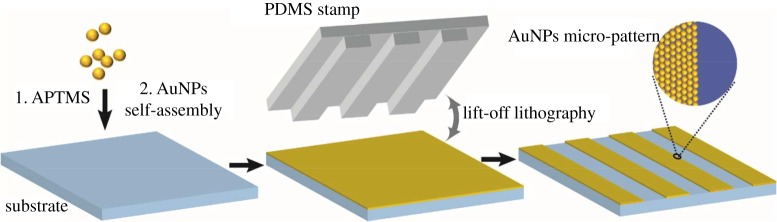
A schematic illustration of the procedure to fabricate a patterned SERS substrate. First SERS substrate with gold nanoparticles is prepared using an APTMS-assisted surface-assembly method; then a polydimethylsiloxane stamp is brought in to make conformal contact with the gold nanoparticle layer; the removal of the PDMS stamp lifts off the gold nanoparticles and leaves the desired SERS-active pattern.

**Figure 1.**
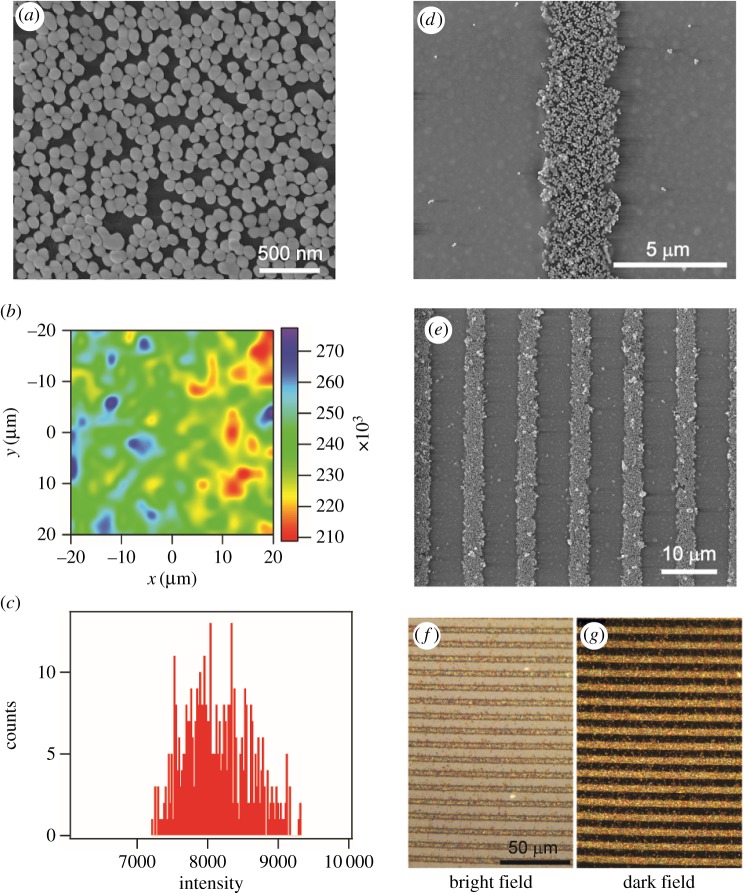
Images of patterned gold nanoparticle arrays on glass slides: (*a*) SEM image of a gold nanoparticle-assembled substrate; (*b*) SERS mapping of the substrate with the SERS peak of 1094 cm^−1^ of 4-mercaptopyridine. The mapping area was 40 × 40 µm^2^ with a step length of 2 µm. The laser power was 0.2 mW and the integration time was 1 s. (*c*) The statistical calculation of all the intensities of the mapping data. (*d*,*e*) SEM images of parallel lines of the two-dimensional gold nanoparticle arrays fabricated by using lift-up lithography, with different magnifications of 20 000× and 5000×, respectively; and (*f*) bright-field and (*g*) dark-field image of the same patterned gold nanoparticle-assembled substrate.

Scheme 1 illustrates the procedure for the fabrication of the gold nanoparticle micro-pattern by lift-up lithography. A high-performance SERS substrate was first prepared on a glass slide as reported with an enhancement factor higher than 1.4 × 10^6^ [18,19]. The SERS uniformity of the AuNP monolayer is shown by SERS mapping using the intensity of a 1094 cm^−1^ peak of pre-adsorbed 4-mercaptopyridine (figure 1*b*,*c*). The difference between the highest and the lowest intensity in all the spectra is 22.7%, which is very close to the requirement of commercial SERS substrates [29]. The relative standard deviation is only 5.6%. The method allows us to fabricate a uniform SERS substrate with a large area. Next, part of the AuNP monolayer on top of the slide is peeled off using a PDMS stamp patterned with microfeatures. A PDMS cover pre-patterned with microchannels is mounted and bonded to the glass slide with the left micro-pattern of nanoparticles. Ultimately, the SERS active zone is encapsulated at the bottom of the microchannels.

The morphology of the micro-patterned gold nanoparticle assembled substrate was characterized by SEM and dark-field microscopy. The SEM images of the substrate (figure 1*a*) show a high density of gold nanoparticles distributed quite uniformly on the substrate. Figure 1*b*,*c* shows the large-area SEM images of the stripe-patterned gold nanoparticle substrate with a stripe width of approximately 4 µm. Only a few nanoparticles were left on the stamp-adhered zone as shown in figure 1*d*,*e*. The mutual validation of the reflective bright-field and dark-field images over the same area of micro-patterned substrate indicates a good spatial uniformity and high assembly density over a large area (figure 1*f,g*). During the lift-up process, the unreacted mobile oligomers on the PDMS surface serves as an adhesive glue [30], and the chemical interaction between the stamp and the nanoparticles is stronger than the electrostatic interaction between the nanoparticles and the substrate, so that the nanoparticles can be lifted up by the stamp.

**Figure 2.**
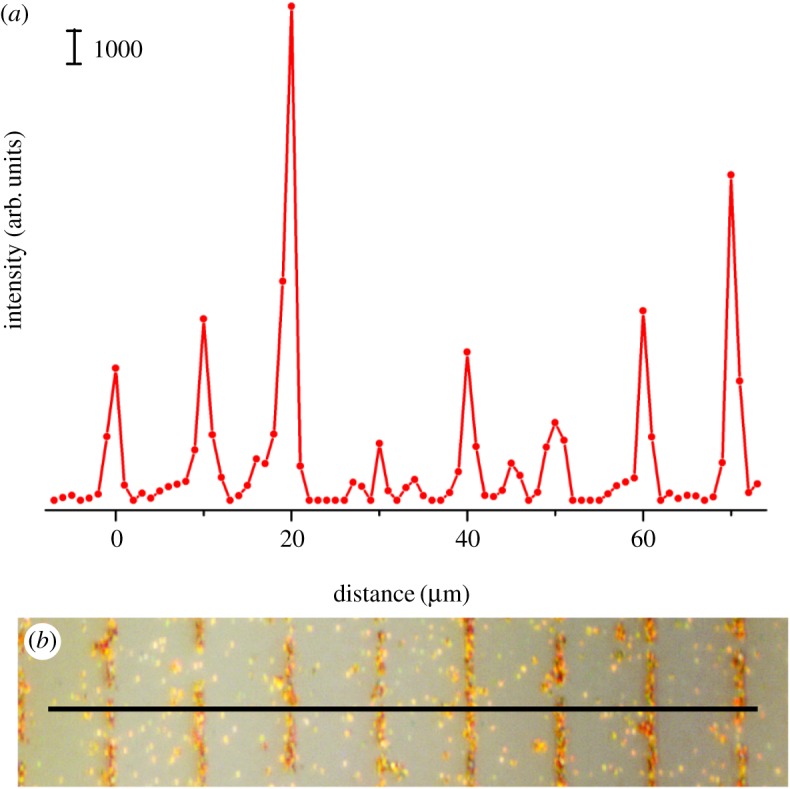
(*a*) SERS signal line scanning on a gold nanoparticle-patterned substrate (80 µm, step length 1 µm) with the Raman peak of 612 cm^−1^ of Rhodamine 6G, and (*b*) optical micrograph of the line scanning region. SERS were excited by a 638 nm laser with a power of approximately 0.1 mW, and acquisition time of 10 s. The laser spot size was approximately 1 µm in diameter.

SERS activity of the patterned substrate was verified by SERS measurement of a probe molecule. For testing the limit of this method, we fabricated very narrow stripes of approximately 2 µm in width by the lift-up process (figure 2*b*), which equals the laser spot size in SERS measurement. The substrate was then incubated with Rhodamine 6G, a dye with a strong Raman signal. Raman line scanning was carried out along the direction perpendicular to the stripe, with a step length of 1 µm. Note that though there were still sparse gold nanoparticles on the stripped regions, they had negligible effects on the SERS because most of them were not closely packed and of low SERS enhancement. Figure 2*a* plots the SERS intensity of a characteristic peak of 612 cm^−1^ along the dark solid line in figure 2*b*. By comparing figure 2*a*,*b*, we can clearly see that the SERS enhancement distribution is consistent with the gold nanoparticle pattern. The average signal is 6164 ± 4429 counts for the gold-nanoparticle pattern area, much higher than the background of 271 ± 359 counts for the gold-nanoparticle stripped area, which indicates that the resulting intact SERS-active regions could be clearly distinguished from the subtractive areas. The large dispersion of signal could be explained as depicting the elastic PDMS stamp deformation squeeze, affecting the nanoparticle structure at the edge of the press and lift-up process. The results indicate that the lift-up strategy has the capacity to reach the resolution limit of SERS mapping (usually equal to the focused laser spot on the substrate).

**Figure 3.**
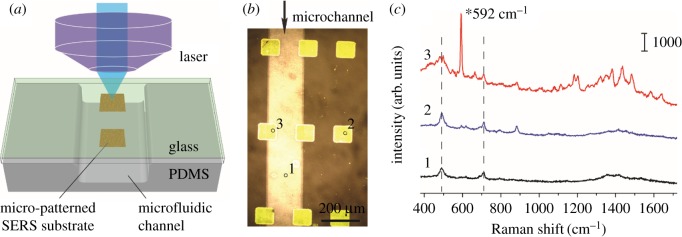
Raman spectra of Nile blue measured at different positions of the microfluidic SERS chip: (*a*) illustration of SERS detection at the SERS-active substrate inside a microfluidic channel; (*b*) an optical microscopic image of the SERS-active substrate (yellow squares) embedded in a microfluidic chip; and (*c*) three SERS spectra measured at three representative positions of the microchannel (*b*), 592 cm^−1^ peak is the characteristic peak of Nile blue. SERS were excited by a 785 nm laser with a laser power of approximately 3 mW and acquisition time of 10 s.

An advantage of the lift-up lithography is that we could fabricate the gold nanoparticle layer with desired shapes, and integrate the SERS-active substrate into a microfluidic system flexibly. We first patterned 100 µm square 10 × 10 arrays of gold nanoparticles on a glass substrate. Next, the PDMS channels were treated with oxygen plasma and mounted onto the glass for bonding (figure 3*a*,*b*). The chip was placed on the reflection Raman microscope with the glass slide facing up to avoid the distortion of the optical path by the thick PDMS cover and solution in the channel. Nile blue solution at a concentration of 1.0 × 10^−4^ M was injected into the channels and incubated for 30 min before flushing out. We collected the SERS spectra from three representative regions. The characteristic Raman peak at 592 cm^−1^ was assigned to the heterocyclic ring in-plane deformation of Nile blue [31], which could be clearly identified from the spectrum on the SERS active substrate (position 3 in figure 3*b*). As a comparison, the SERS signals of Nile blue were negligible, both on the channel region free of gold nanoparticles (position 1 in figure 3*b*) and the SERS-active region but bounded to the PDMS cover.

The main challenge of performing SERS measurement in a microfluidic SERS chip compared with that on a planar SERS substrate is how to collect satisfactory Raman signals in the microchannels. The influence of PDMS is an important issue of concern. The Raman peaks at 489 cm^−1^ (Si–O–Si stretching) and 708 cm^−1^ (Si–C symmetric stretching) are assigned to the bulk PDMS (figure 3*c*) [32]. However, in the present design, the SERS spectra were collected from the glass slide, and the distortion of the optical path by the bulk PDMS and fluidic layer was minimized. Furthermore, the intrinsic background of PDMS was suppressed, making the SERS signal of the target molecule distinct enough for highly sensitive detection.

**Figure 4.**
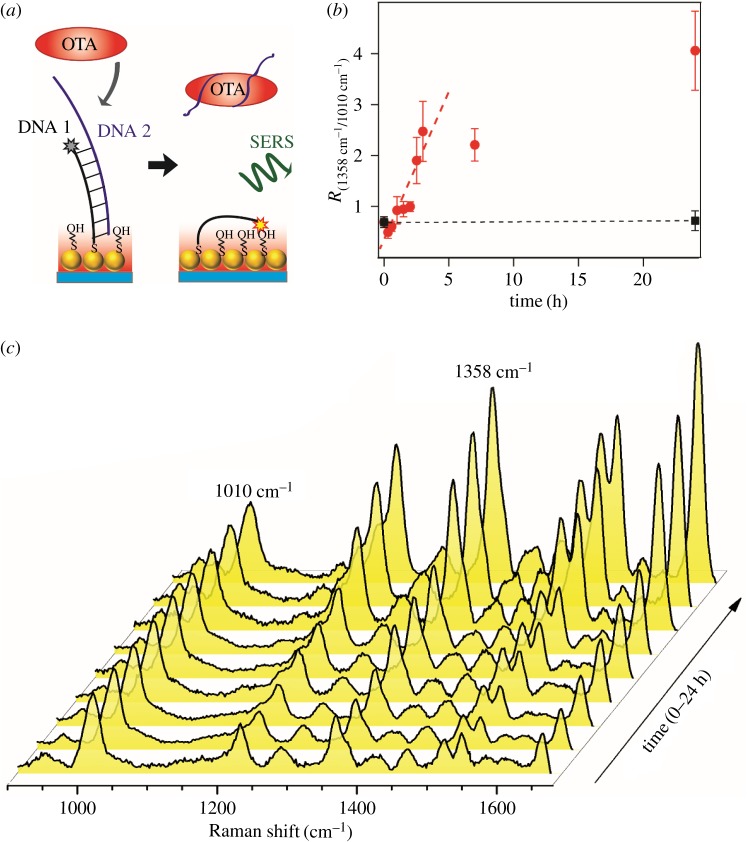
(*a*) Schematic illustration of the SERS detection of OTA using OTA aptamer in the microfluidic SERS chip. (*b*) Relative intensity of 1358 cm^−1^/1010 cm^−1^ as a function of the incubation time with an OTA concentration of 1 µM (red circle) and without OTA as a control (black square), respectively. The data points represent the average and the s.d. of 16 spectra from different locations. (*c*) Evolution of average SERS spectra with incubation time; the spectra were baseline corrected and normalized with respect to the peak of 1010 cm^−1^.

Optofluidic SERS detection of biomolecules was performed on the SERS-active surface (figure 4). In this section, ochratoxin A (OTA) was employed as the target molecule. OTA is a mycotoxin that has been detected in different food products, which may cause potential public health risks [33–36]. The specific detection of OTA was accomplished by an OTA-binding aptamer (figure 4*a*): the OTA aptamer (DNA 2) and complementary strand labelled with TAMRA (DNA 1) were hybridized to form a stiff double-stranded structure, which was immobilized on the SERS-active pattern via a S–Au bond. In the presence of OTA, OTA can interact with the aptamer, which leads to the dissociation of the DNA double strand. The remaining DNA 1 on the substrate becomes flexible and the reporter molecule (TAMRA) can approach closer to the surface. Thus, an amplification of the SERS signals of the reporter will be observed due to a decrease in the distance of the reporter to the gold surface. In the absence of OTA, the DNA duplex adopted a rigid conformation perpendicular to the gold surface and the signal is weaker. Figure 4*c* shows the evolution of average SERS spectra with incubation time at a fixed OTA concentration of 1 µM. The spectra are displayed after subtracting the background and normalized with respect to the peak of 1010 cm^−1^. The SERS peaks at 1080 cm^−1^ correspond to C–C stretch deformation of the spacer molecules of mercaptoethanol. With the incubation of OTA, the intensities of those SERS peaks at 1217, 1358, 1510, 1535 and 1649 cm^−1^ (assigned to TAMRA) increased with time, indicating that the TAMRA bent over and became closer to the hot spots in colloidal nanoparticles following the release of aptamer DNAs. The relative intensity of 1358 cm^−1^/1010 cm^−1^ was monitored to show the dissociation rate of the DNA duplex (figure 4*b*). The intensity ratio revealed no significant difference after 1 day of incubation without OTA, showing the stability of the double-stranded DNA. When incubated with 1 µM OTA, the intensity increased at the beginning, indicating the dissociation of the DNA duplex and the binding of aptamer and OTA. The signal reached a plateau after 3 h, implying the saturation for the aptamer and OTA complex. However, it should be noted that the signal ratio variation did not show concentration-dependent change at a 500 nM OTA concentration and lower, which means the sensitivity so far could not meet the food safety requirement (approx. 5 nM). The following reason may account for that: the double-stranded DNA is not rigid enough for this distance-sensitive detection strategy. If appropriate, a structure with the Hoogsteen base pairs [37] or quadruplex [38] will be more practical to achieve a better performance.

## 4. Conclusion

To conclude, this work demonstrates a successful approach to develop a SERS substrate-embedded microfluidic chip by using facile lift-up lithography with good reproducibility. The SERS performance of the fabricated micro-patterned substrate was evaluated by using R6G as the SERS probe. Optofluidic devices were further fabricated for *in situ* detection of desired SERS signal of Nile blue in the microchannel, with the limited distraction of impurities and background of bulk PDMS. Finally, the indirect SERS detection of OTA, a mycotoxin binding with aptamer, was successfully demonstrated. These results suggest that the lift-up lithography approach enables simple, feasible and large-scale implementation of a high-performance planar SERS substrate with microfluidic networks.

The integration of microfluidics and SERS is of particular interest for the detection of any trace target molecules interacting with the SERS substrate with a limited solution quantity. The SERS structures could be easily reduced in size in the range of microchannel width without any complicated fabrication and special instruments. It could be expected that the cost-effective SERS chips would be promising for disposable applications in field examination, such as monitoring water pollution, food safety inspection and so on.

## Supplementary Material

supplemental results for R6G spectra and OTA titration

## Data accessibility

The data supporting this article are available in the electronic supplementary material. All the datasets are available at Dryad (https://doi.org/10.5061/dryad.g3q80) [39].
