# Supplementary material for: Facile fabrication of microfluidic surface-enhanced Raman scattering devices via lift-up lithography
Source: R Soc Open Sci. 2018 Apr 4;5(4):172034. doi: 10.1098/rsos.172034 (PMC5936922; doi:10.1098/rsos.172034)
Supplement: supplemental results for R6G spectra and OTA titration [file rsos172034supp1.docx]

Supporting Information

Facile Fabrication of Microfluidic Surface-Enhanced Raman Scattering Devices via Lift-up Lithography

Yuanzi Wu,^a^ Ye Jiang,^a^ Xiaoshan Zheng,^b^ Shasha Jia,^b^ Zhi Zhu,^b^ Bin Ren*^b^ and Hongwei Ma*^c^

a. College of Biological Science and Engineering, Fuzhou University, Fuzhou 350002, China.

b. State Key Laboratory of Physical Chemistry of Solid Surfaces, the MOE Key Laboratory of Spectrochemical Analysis & Instrumentation, College of Chemistry and Chemical Engineering, and Collaborative Innovation Center of Chemistry of Energy Materials, Xiamen University, Xiamen 361005, China. E-mail: bren@xmu.edu.cn. Tel.: +8605922186532

c. Suzhou Institute of Nano-Tech and Nano-Bionics, Chinese Academy of Sciences, Suzhou 215125, China. E-mail: hwma2008@sinano.ac.cn. Tel.: +86051262872539





Figure S1. Typical Raman spectra of Rhodamine 6G adsorbed on the AuNP assembled area (c), and AuNP stripped area (a) in Figure 2. (b) SERS spectrum of pristine AuNP assembled area.





Figure S2. The relative intensity of 1358 cm^-1^/1010 cm^-1^ as a function of the concentration of OTA ranging from 1 to 500 nM. Means and error bars are calculated from 16 spectra from different locations.





Figure S3. 10 raw spectra for SERS mapping of 4-mercaptopyridine adsorbed on the SERS substrate.

Surface enhancement factor (G) is calculated by the following equation:^1^

$$G=\frac{{I_{surf}}/{N_{surf}}}{{I_{bulk}}/{N_{bulk}}}$$

Where *I_surf_* and *I_bulk_* correspond to the Raman intensity for the strongest peak of the surface and solution species, respectively. *N_surf_* and *N_bulk_* correspond to the number of the molecules which are effectively excited by the laser beam on the surface and in the solution, respectively. *N_surf_* can be estimated by simplifying the molecules adsorption as less than a monolayer on the surface of closed packed nanospheres.

$$N_{surf}=\frac{RA}{\sigma}$$

Where R is the specific area of the nanoparticles, A is the area of the focal spot of the laser, σ is the surface area occupied by a single molecule.

*N_bulk_* is calculated by

$$N_{bulk}=AhcN_{A}$$

Where *c* is the analyte concentration, *N_A_* is the Avogadro constant. h (in micrometers) is the confocal depth of laser in the solution.

1 Ren B, Liu GK, Lian XB, Yang ZL, Tian ZQ. 2007 Raman spectroscopy on transition metals. Anal. Bioanal. Chem. 388, 29-45
